# A question of scale

**DOI:** 10.7554/eLife.50890

**Published:** 2019-09-10

**Authors:** Muireann Irish, Siddharth Ramanan

**Affiliations:** 1 Brain and Mind Centre and School of PsychologyUniversity of SydneyNew South WalesAustralia; 2 ARC Centre of Excellence in Cognition and its DisordersMacquarie UniversityNew South WalesAustralia

**Keywords:** spatial scale, default-mode network, cortical gradient, PPA, RSC, OPA, Human

## Abstract

An fMRI experiment reveals distinct brain regions that respond in a graded manner as humans process distance information across increasing spatial scales.

**Related research article** Peer M, Ron Y, Monsa R, Arzy S. 2019. Processing of different spatial scales in the human brain. *eLife* **8**:e47492. doi: 10.7554/eLife.47492

Just like our ancestors before us, humans must be able to navigate within both familiar and new environments, whether this involves driving to work or finding our way around a new city. Successful spatial navigation depends on many cognitive processes including memory, attention, and our perception of direction and distance (Epstein et al., 2017). A key issue, however, is that spatial environments vary considerably in terms of their size and complexity. To date most research on spatial navigation has focused on small spatial scales, such as navigating within a room or a building (Wolbers and Wiener, 2014). But it remains unclear how accurately we can estimate distances between locations on a larger scale, such as whether the Taj Mahal is closer to the Pyramids of Giza or the Great Wall of China, and how these different spatial scales are represented in the brain.

Now, in eLife, Michael Peer, Yorai Ron, Rotem Monsa and Shahar Arzy – who are based at the Hebrew University of Jerusalem, the Hadassah Medical Center and the University of Pennsylvania – report a simple but elegant experiment that teases apart which brain regions are recruited when we process information about environments that are on different spatial scales (Peer et al., 2019). Peer et al. asked internationally-travelled adults to provide the names of two locations they were personally familiar with across six spatial ‘scales’. These scales varied from small, spatially-confined areas (e.g. rooms and buildings) through medium-sized regions (e.g. local neighborhoods and cities) to expansive geographical locations (e.g. countries and continents; Figure 1A). The experiment was then personalized by asking each participant to provide the names of eight items that were personally familiar to them within each location.

**Figure 1.**
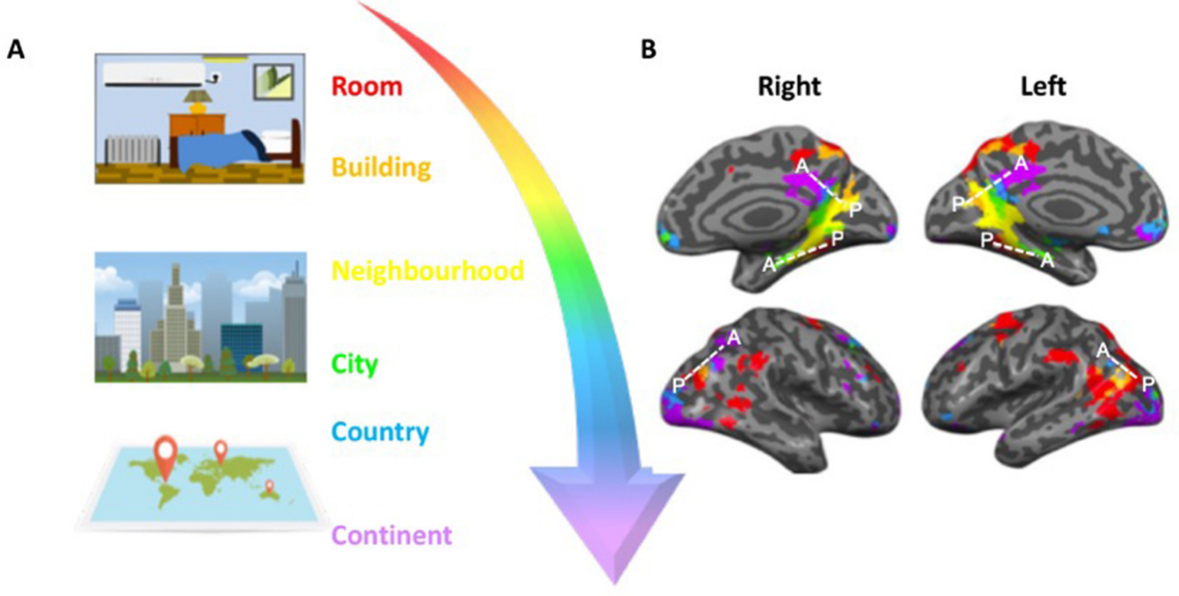
How different spatial environments are represented in the human brain. (**A**) In order to navigate successfully humans must be able to judge distances between objects on both small (e.g. rooms and buildings) and large (e.g. cities and countries) scales. (**B**) Peer et al. showed that estimating distance across different spatial scales engages three main clusters of brain regions that are organized along a gradient (represented by the white dashed lines in each hemisphere). Within each cluster, spatial environments that are smaller and more constrained (red and orange) are represented in posterior portions, whilst larger, less-constrained environments (blue and purple) are represented in more anterior portions of the clusters. The middle surface of the brain (where the right and left hemispheres meet) is shown in the upper panels; the outer surfaces of the two hemispheres are shown below.

Image credit: Adapted from Peer et al. (2019).

A few days later, participants underwent a functional magnetic resonance imaging experiment to determine which areas of the brain are selectively involved during spatial processing. This technique enables researchers to measure increases in blood flow and oxygen delivery to parts of the brain, and determine which regions are more ‘active’ when engaging in a cognitive task. During the experiment, participants were asked to judge distances between a ‘target’ item from their personal list (e.g. a table in their bedroom) and two other items from the same location (e.g. a chair or a bed in their bedroom). This allowed Peer et al. to investigate which brain regions respond to small, medium, and large spatial scales, and which regions are insensitive to scale but respond to other location or proximity information.

The experiment identified three main clusters of brain regions that are important for processing different spatial scales. What was unique about all three clusters was that activity within them shifted in a ‘graded’ manner depending on whether participants were processing spatial information on a local or more global scale. For example, when participants judged distances on a small scale in local environments, this engaged the posterior portions of all three clusters. On the other hand, when participants judged distances on a larger scale, the pattern of activity shifted towards the anterior portions of the clusters (Figure 1B).

These findings align remarkably well with previous work showing that the human hippocampus – a region of the brain involved in spatial navigation (Burgess et al., 2002) – represents object position and spatial information, such as direction and distance between objects, as a graded pattern of activity (Evensmoen et al., 2015; Evensmoen et al., 2013). The latest study, however, extends our understanding by highlighting how graded patterns of activity move from posterior to anterior regions of the spatial processing network outside of the hippocampus, depending on the spatial scale being processed (Figure 1).

The work presented here provides new insights into how humans navigate within different environments. From a clinical perspective, appreciating how humans dynamically zoom in or out of different spatial scales could help refine how various neurological conditions are diagnosed. This is most relevant for neurodegenerative disorders, such as Alzheimer’s disease, in which disorientation and a distorted sense of direction are often early symptoms (Coughlan et al., 2018; Tu et al., 2015). Whether the altered sense of direction and difficulties in judging proximity that are associated with Alzheimer’s disease are due to changes in the way that regions of the brain represent spatial scale is an important question for future studies to address.
